# Senolytic compounds reduce epigenetic age of blood samples in vitro

**DOI:** 10.1038/s41514-025-00199-z

**Published:** 2025-02-04

**Authors:** Vithurithra Tharmapalan, Miriam Du Marchie Sarvaas, Michael Bleichert, Martina Wessiepe, Wolfgang Wagner

**Affiliations:** 1 Institute for Stem Cell Biology, RWTH Aachen University Medical School, 52074 Aachen, Germany; 2 Helmholtz-Institute for Biomedical Engineering, RWTH Aachen University Medical Faculty, 52074 Aachen, Germany; 3 Institute for Transfusion Medicine, Faculty of Medicine, RWTH Aachen University, Aachen, Germany; 4 Center for Integrated Oncology Aachen Bonn Cologne Düsseldorf (CIO ABCD), Cologne, Germany

**Keywords:** Senescence, Business strategy in drug development, Epigenetics, Biomarkers

## Abstract

Senolytic drugs raise the expectation that they can specifically eliminate a subset of senescent cells in a given tissue. In this study, we have exemplarily analyzed if a 3-day treatment of human blood samples in vitro would reduce age-associated biomarkers, with a particular focus on epigenetic age-predictions. Of eight tested compounds, JQ1, RG7112, nutlin-3a, and AMG232 reduced epigenetic age, indicating that this approach may be useful in drug screening for senolytic compounds.

## Introduction

Cellular senescence is a state of permanent cell cycle arrest, where cells remain metabolically active but cease to proliferate or enter apoptosis^1^. While senescence may contribute to prevent tumor formation, growing evidence suggests that the accumulation of senescent cells negatively impacts tissue function as we age^2^. Small-molecule compounds have been identified to selectively target senescent cells. These so-called senolytic drugs may provide promising strategies to prevent age-associated conditions^3^.

To identify novel and more effective senotherapeutic drugs and combinations, high-throughput screening methods have been employed. For instance, one approach involves the detection of senescence-associated β-galactosidase (SA-β-gal) in murine embryonic fibroblast^4^. Alternatively, high-throughput screening has been used for the detection of p16^Ink4a^-positive cells in vitro and for phenotypic screening in vivo^5^. So far, in vitro screening of senolytic compounds mainly relied on fibroblasts, with only a few studies considering peripheral blood mononuclear cells (PBMCs). Furthermore, little is known about how senolytic drugs impact epigenetic age predictions.

Aging is associated with highly reproducible DNA methylation changes (DNAm), which can be leveraged for age-prediction^6,7^. While many of these predictions are based on genome-wide epigenetic profiles, targeted analysis of a small number of age-associated CpGs has also been shown to yield accurate results^8,9^. We anticipated that senescent subsets exist within leukocytes, and if senolytic drugs preferentially target these cells, they might also reduce epigenetic age estimates.

## Results and discussion

We selected eight compounds with potential senolytic activity that target different anti-apoptotic pathways: Among these, RG7112 (RO5045337)^10^, AMG232 (KRT-232; Navtemadlin)^11^, and nutlin-3a^12^ are inhibitors of the nuclear-localized E3 ubiquitin ligase MDM2, which disrupt the interaction between p53 and MDM2, leading to a stabilized p53 and promoting apoptosis in senescent cells. JQ1 is a BET bromodomain inhibitor that targets the non-homologous end joining DNA repair pathway^13^. We also included BH3 mimetics such as ABT263 (navitoclax), which targets multiple BCL-2 family proteins^14^, and S63845, which specifically inhibits the anti-apoptotic protein MCL-1^15^. Additionally, we used the tyrosine kinase inhibitor dasatinib and the naturally occurring flavonoid quercetin, which inhibits the SIRT1/PI3K signaling pathway, often used in combination for synergistic effects^16^. Lastly, piperlongumine, an amide alkaloid of the fruit of long pepper that selectively induces senescent cell death by inhibiting oxidative stress response proteins, was included^17^. To determine the half maximal inhibitory concentration (IC_50_) of these compounds, we have treated PBMCs of healthy donors during three days of in vitro culture with different concentrations and analyzed viability (Supplemental Fig. S1). Based on these results, we selected two concentrations—one below and one above the IC50 (subsequently referred to as low and high, respectively)—to evaluate their effects on epigenetic age predictions in 12–18 donors of different ages (22–68 years). The aging signature used for these predictions was based on three CpGs in the genes *CCDC102B, FHL2*, and *PDE4C*^18^. In untreated PBMCs, the correlation between predicted and chronological age was strong (*R*^2^ = 0.95) with a mean age deviation of 0.34 years. Furthermore, repeated measurements confirmed a very high reproducibility of these measurements (Supplemental Fig. S2).

**Fig. 1:**
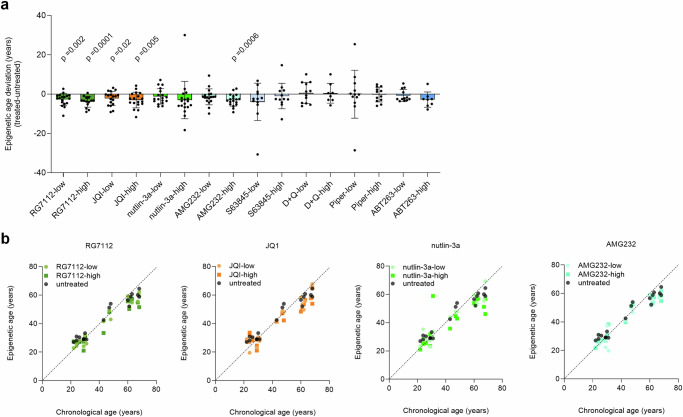
Treatment of leukocytes with senolytic compounds affects epigenetic age. **a** Peripheral blood mononuclear cells (PBMCs) of healthy donors (*n* = 12–18) were cultured for 3 days with eight different compounds at concentrations below and above IC_50_ (low and high, respectively): RG7112, JQ1, nutlin-3a, AMG232, S63845, dasatinib in combination with quercetin (D + Q), piperlongumine (Piper), and ABT263. Epigenetic age was determined with and without treatment and the deviation is depicted here. One sample *t*-test was used to estimate significance. **b** For the four compounds that revealed a decline in epigenetic age, the epigenetic age predictions are plotted against the chronological age of the donors (*n* = 18), demonstrating that the decline was observed across all age-groups.

Remarkably, treatment with four compounds led to a decrease in epigenetic age predictions: RG7112 (average deviations of −2.7 and −3.9 years for low and high concentrations, respectively; one-sample *t*-test of age-deviations: *p* = 0.002 and 0.0001), JQ1 (average deviations of −2.2 and −3.0 years; *p* = 0.02 and 0.005), and AMG232 (average deviation for high = −3.0 years; *p* = 0.0006). Furthermore, a clear tendency was observed for nutlin-3a (average deviations of −1.2 and −3.0 years), although this did not reach statistical significance. For the other drugs, we did not observe a clear effect on epigenetic age-predictions (Fig. 1). Similar results were observed by paired *t*-test of absolute epigenetic age-predictions (corresponding controls versus treated samples; Supplemental Fig. S3).

It’s important to note that the epigenetic aging signatures were not specifically trained for the state of cellular senescence, but rather for chronological age. We have previously demonstrated that DNAm changes during replicative senescence and aging have rather moderate overlap^19,20^ and several further studies have also shown that cellular senescence and epigenetic aging have independent mechanisms^21–23^. Nonetheless, our findings suggest that certain senolytic compounds reduce epigenetic age estimates, possibly due to preferential targeting of more senescent cells from PBMCs.

**Fig. 2:**
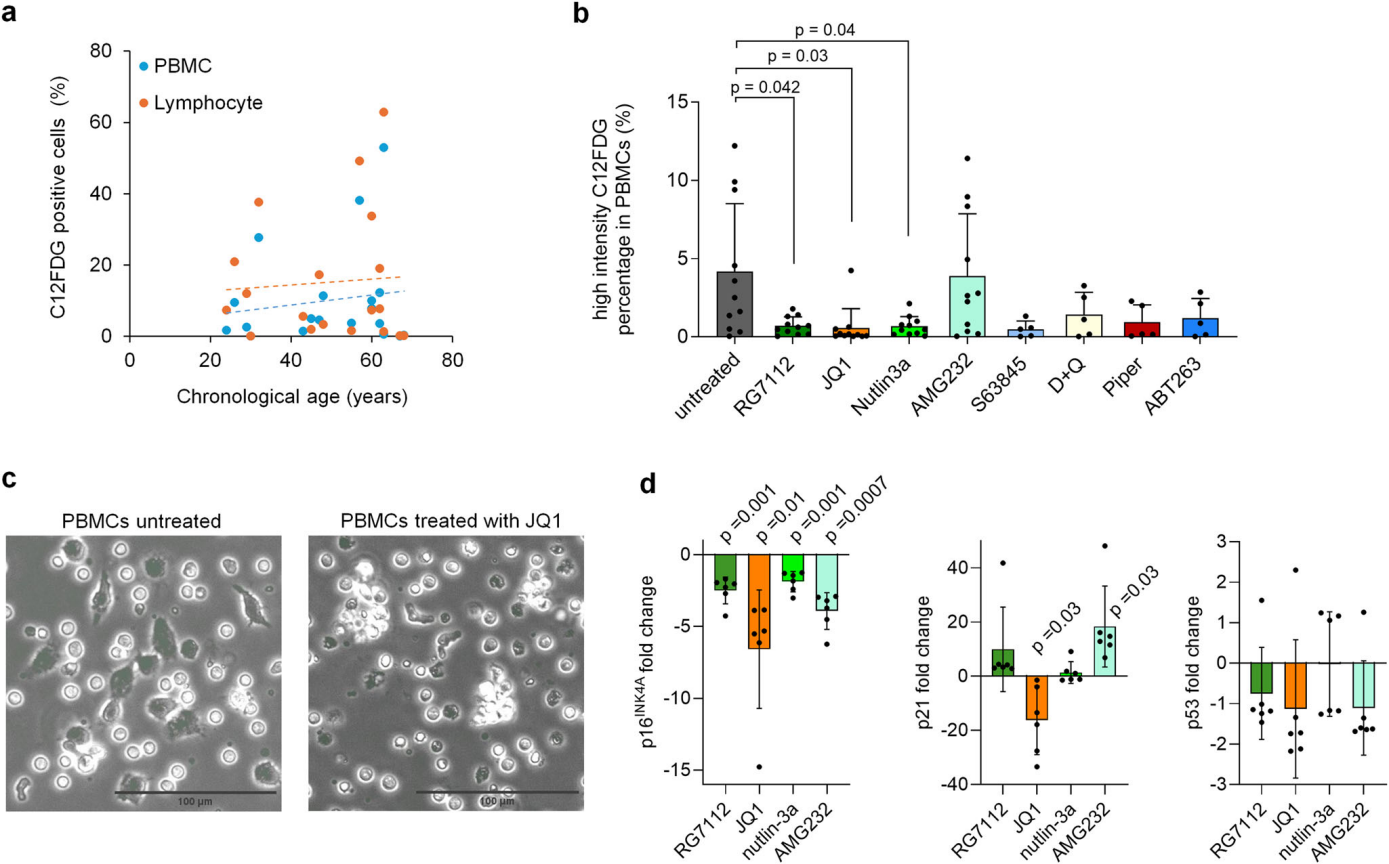
Senolytic treatment affects also other senescence-associated biomarkers. **a** Staining of senescence-associated β-galactosidase (SA-β-gal) with C12FDG revealed in tendency a slight increase of positive cells with aging. Flowcytometric analysis of either peripheral blood mononuclear cells (PBMCs) or the lymphocytes of healthy donors (*n* = 19). **b** In analogy, PBMCs were cultured for 3 days with RG7112 (10 µM), JQ1 (10 µM), nutlin-3a (10 µM), AMG232 (1 µM), S63845 (1 µM), dasatinib in combination with quercetin (D + Q; 20 nM + 20 µM), piperlongumine (Piper; 10 µM), and ABT263 (200 nM). *n* = 5 and for the four candidate compounds that revealed effects on epigenetic age *n* = 11; Two-way ANOVA with multiple comparisons was performed to assess statistical significance. **c** Exemplary phase contrast images of C12FDG stained PBMCs without or with treatment with JQ1 for 3 days. Upon treatment, there were less irregularly shaped large plastic adherent cells. **d** RT-qPCR analysis of senescence-associated gene expression of p16, p21, and p53 upon treatment with RG7112, JQ1, nutlin-3a, and AMG232. One sample *t*-test was used to assess statistical significance.

To better assess the heterogeneity of senescence, we stained SA-β-gal with 5-dodecanoylaminofluorescein di-β-d-galactopyranoside (C12FDG) and analyzed the proportion of positive cells either in all PBMCs or the lymphocyte compartment (Supplemental Fig. S4a, b). We observed a moderate age-related increase in C12FDG-positive cells in both PBMCs and lymphocytes (Fig. 2a). This trend aligns with a recent study showing that the percentage of circulating C12FDG-positive PBMCs correlates with aging and serum biomarkers associated with senescence^24^. Following treatment with the eight senolytic compounds, we noted a significant reduction in C12FDG positive cells, particularly in PBMCs with RG7112, JQ1, and nutlin-3a (Fig. 2b). A similar, though less pronounced, effect was also observed in lymphocytes (Supplemental Fig. S4c). Flowcytometric analysis of the lymphocyte compartment revealed that the senolytic compounds shifted the balance of NK cells and B cells towards T cells (Supplemental Fig. S4d). However, in vivo, treatment with JQ1 has been shown to decrease B cells and T cells^25^. Phase contrast microscopy indicated that senolytic treatment preferentially targets larger, irregularly shaped plastic adherent cells (Fig. 2c). We also examined how gene expression of *CDKN2A* (p16^INK4a^), *CDKN1A* (p21) and *TP53* (p53) was influenced by the four senolytic compounds that affected epigenetic age predictions. Notably, p16^INK4a^ expression decreased consistently across all four treatments, while changes in p21 and p53 were less consistent (Fig. 2d). In summary, our findings suggest that RG7112, JQ1, AMG232, and nutlin-3a preferentially target the senescent subset of PBMCs in vitro.

Relying on a single marker or measuring cellular senescence can lead to misleading conclusions. For instance, increased SA-β-gal staining might not solely indicate senescence but could also reflect elevated lysosomal mass and autophagic activity, potentially capturing quiescent cells with reversible growth arrest^26^. Furthermore, a recent comparison of colorimetric (X-Gal) and fluorescent (C12FDG) SA-β-Gal staining demonstrated only a moderate correlation, indicating that the two approaches are not trivially interchangeable^27^. Our results indicate that epigenetic age-predictions may complement the search for senolytic compounds. Treatment with RG7112, JQ1, AMG232, and nutlin-3a not only reduced epigenetic age predictions but also decreased SA-β-gal staining and p16^INK4A^ expression. However, for those samples that were tested for senescence markers and epigenetic age, we did not observe a clear correlation. Thus, it is still not proven if epigenetic rejuvenation is due to the removal of senescent cells.

The underlying mechanisms driving epigenetic clocks remain unclear. Some studies suggest that stochastic epigenetic modifications accumulate with age^28,29^, while others propose that age-related patterns are co-regulated within cells^30,31^. This raises the possibility that epigenetic age predictions in bulk PBMCs might reflect a mix of younger and older cells, a notion supported by observations that malignant diseases appear to inherit aspects of epigenetic age from the tumor-initiating cell^18^. Furthermore, epigenetic age-predictions vary in different leukocyte subsets^32^, and therefore we cannot exclude that epigenetic rejuvenation is partially attributed to moderate changes in cell composition. Although our proof of concept study is limited in sample size, it demonstrates the feasibility of using epigenetic age predictions in screening approaches. In this context, targeted age-predictors, such as the three CpGs utilized in this study, are more cost-effective and practical compared to genome-wide approaches^9^. A recent study using the combination treatment of dasatinib and quercetin in vivo demonstrated inconsistency between different generations of epigenetic clocks that were based on genome-wide DNAm profiles^33^, and we did not observe epigenetic rejuvenation with this combination, either. This underscores the urgent need to develop new biomarkers for quantifying senescence and aging to apply them for in vitro and in vivo applications. Moreover, the effects of senolytic compounds on age-associated biomarkers do not necessarily indicate their safety and effectiveness in slowing organismal aging, which still requires thorough investigation.

## Materials and methods

### Blood samples

Peripheral blood samples from 19 healthy blood donors were used for the study. All samples were collected from the Department of Transfusion Medicine with informed and written consent, in compliance with the Declaration of Helsinki, and the research was specifically approved by the local ethics committee of RWTH Aachen University (EK 041/15). Peripheral blood mononuclear cells (PBMCs) of healthy donors were isolated by gradient centrifugation with Pancoll (Pan Biotech, Aidenbach, Germany).

### Senolytic compounds

We selected eight different senolytics based on their distinct apoptotic mechanisms and their ability to target anti-apoptotic proteins, which are known to induce apoptosis in senescent cells: RG7112 (Selleck Chemicals, Houston, USA), JQ1 (Sigma-Aldrich, St Louis, USA), nutlin-3a (Selleck Chem), AMG232 (Axon medchem, Groningen, Netherlands), S63845 (Selleck Chem), dasatinib (Selleck Chem) in combination with quercetin (D + Q; Sigma-Aldrich), piperlongumine (Selleck Chem), and ABT263 (Selleck Chem). These compounds were supplemented for the growth media for PBMCs, consisting of StemSpan Serum-Free Expansion Medium (Stemcell Technologies, Vancouver, Canada), with 10 ngc stem cell factor (SCF), 20 ng/mL thrombopoietin (TPO), 10 ng/mL fibroblast growth factor 1 (FGF-1, all PeproTech, Hamburg, Germany), 10 μg/mL heparin (Ratiopharm, Ulm, Germany), and 100 U/mL penicillin/streptomycin (Lonza, Basel, Switzerland). Cell viability was tested after 3 days with Cell Titer-Glo 2.0 luminescent cell viability assay (Promega, Wisconsin, USA) in 96-well plates (10,000 cells/well; 3 wells per condition), using BioTek Synergy 2 plate reader and Gen5 software (Agilent Technologies, California, USA) to determine IC_50_ curves. The following concentrations below (low) and above (high) the IC_50_ were then selected: RG7112 (10, 50 µM), JQ1 (10, 20 µM), nutlin-3a (10 and 50 µM), AMG232 (1 and 10 µM), S63845 (500, 1 µM), dasatinib (D) combined with quercetin (Q) (20 nM D + 50 nM Q, 20 µM D + 50 µM Q, both), piperlongumine (10, 50 µM), and ABT263 (200, 500 nM).

### Epigenetic age prediction with pyrosequencing

DNA was isolated from 500,000 cells per well using the QIAamp DNA Mini Kit (Qiagen, Hilden, Germany). DNA was quantified with a Nanodrop 2000 Spectrophotometer (Thermo Scientific, Wilmington, USA), and bisulfite was converted with the EZ DNA Methylation Kit (Zymo Research, Irvine, USA). To determine epigenetic age, we measured the three age-associated CG dinucleotides (CpG sites) linked to the coiled-coil domain-containing protein 102B (*CCDC102B*), four and a half LIM domains protein 2 (*FHL2*), and phosphodiesterase 4C (*PDE4C*). Primers and protocols for pyrosequencing were previously described in earlier studies^9,18^. PCR was performed using the PyroMark PCR kit (Qiagen), and pyrosequencing was then performed on the PyroMark Q48 Autoprep system (Qiagen) using the PyroMark Q48 Advanced Reagent Kit. The results were analyzed using PyroMark Q48 Advanced software. Epigenetic age was calculated as follows:

$$\begin{array}{l}{\rm{Predicted}}\; {\rm{age}}\,({\rm{in}}\;{\rm{ years}})=3.86+0.825{{\rm{DNAm}}}^{{\rm{FHL}}2}\\-\,0.342{{\rm{DNAm}}}^{{\rm{CCDC}}102{\rm{B}}}+\,1.177{{\rm{DNAm}}}^{{\rm{PDE}}4{\rm{C}}}\end{array}$$

Epigenetic age deviation was calculated by subtracting the predicted age of corresponding controls from each treated sample.

### Senescence-associated beta-galactosidase assay

We employed SA-β-gal staining as a surrogate marker for senescence. In brief, PBMCs (*n* = 19) were pre-treated with 100 nM bafilomycin A1 (MedChemExpress, Monmouth Junction, USA) for one hour and then treated with 33 µM 5-dodecanoylaminofluorescein di-β-d-galactopyranoside (C12FDG; Abcam, Cambridge, UK) for 2 h at 37 °C^34^. Cells were washed with PBS + 2% FCS and measured by flow cytometry using a FACS Canto II (BD Bioscience, Franklin Lakes, USA) and analyzed using FlowJo software, version 10.4.2 (BD).

### Immunophenotypic analysis

The following antibodies were used to analyze PBMCs treated with senolytics after 3 days in culture: CD34-APC (clone 581), CD45-V500 (clone HI30), CD3-FITC (clone SK7), CD56-PE-Cy7 (B159) and CD19-PE (4G7; all BD). Antibody staining was conducted in MACS-buffer solution (PBS with 2% FCS and 2 mM EDTA), with incubations performed on ice for at least 30 min. After staining, cells were washed with MACS buffer, centrifuged, and the pellets were resuspended in 200 µL MACS buffer for analysis using the FACS Canto II and FlowJo software, version 10.4.2 (BD).

### Gene expression analysis

To estimate gene expression of three senescence-associated genes (p16, p21, and p53), RNA was isolated from 500,000 cells per well with the NucleoSpin RNA, Mini Kit (Macherey-Nagel, Düren, Germany). RNA was quantified with a Nanodrop 2000 Spectrophotometer (Thermo Scientific, Wilmington, USA). A total of 250 ng of RNA was used to synthesize cDNA using the High-Capacity cDNA Reverse Transcription Kit (Applied Biosystems, Waltham, USA). Quantitative real-time (qRT)-PCR was performed using TaqMan gene expression master mix (Applied Biosystems) and gene-specific TaqMan assays (all from Applied Biosystem) in a StepOnePlus machine (Applied Biosystems). The PCR conditions were 50 °C for 2 min, 95 °C for 10 min, followed by 40 cycles at 95 °C for 15 s and 60 °C for 1 min. TaqMan assays for p16 (HS_00923894_m1), p21 (HS_00355782_m1) and p53 (HS_01034249_m1) were analyzed. The target gene mRNA levels were normalized to the housekeeping gene *GAPDH* (Hs02758991_g1).

### Statistics

One-sample *t*-test was used to determine whether the mean value of the epigenetic age deviations within the group differed significantly from zero (calculated with GraphPad Prism 10). In all other cases, a two-way ANOVA was used to compare untreated and treated samples (calculated with GraphPad Prism 10).

## Supplementary information

Supplemental figure S1-S4

## Data Availability

All data generated or analyzed during this study are included in this manuscript. Any additional materials are available from the corresponding author upon request.
