# Supplementary material for: Senolytic compounds reduce epigenetic age of blood samples in vitro
Source: NPJ Aging. 2025 Feb 4;11(1):6. doi: 10.1038/s41514-025-00199-z (PMC11794651; doi:10.1038/s41514-025-00199-z)
Supplement: Supplementary file 1 — Supplemental figure S1-S4 [file 41514_2025_199_MOESM1_ESM.pdf]

Supplemental Information

Senolytic compounds reduce epigenetic age of blood samples *in vitro*

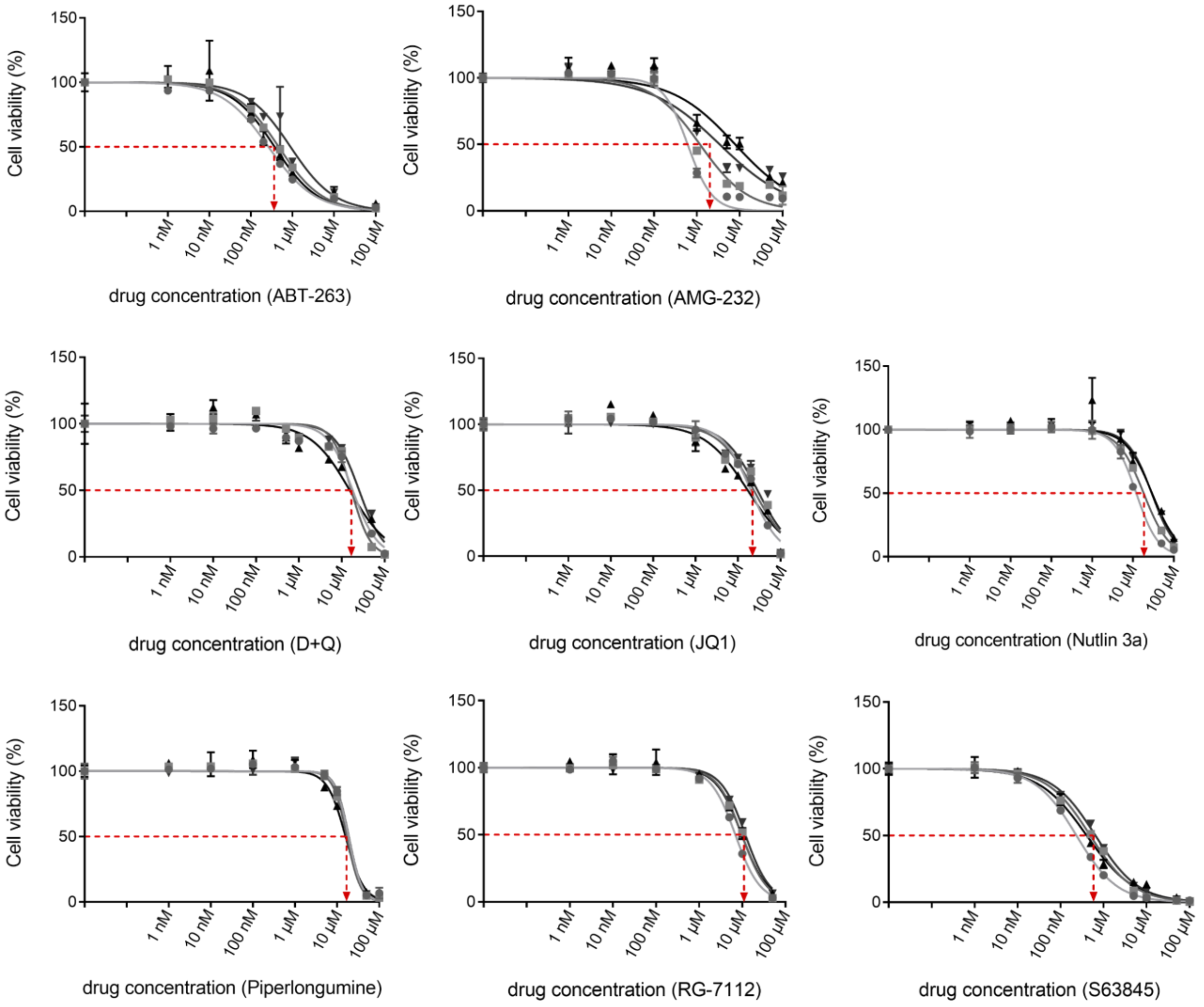

Figure S1: Dose-response curves upon treatment with eight senolytic compounds.

Peripheral blood mononuclear cells of four donors (24, 31, 48 and 68 years) were treated with different concentrations of the senolytic compounds (in 96-well plates, each with three technical replicates). After three days, viability was determined using CellTiter Glo assay. Median IC50 values were determined for each drug: ABT263 = 417.7 nM, AMG232 = 3.26 µM, D+Q = 17.4 µM, JQ1 = 21.75 µM, nutlin-3a = 23.5 µM, piperlongumine = 17.8 µM, RG7112 = 9.67 µM, and S63845 = 446.9 nM.

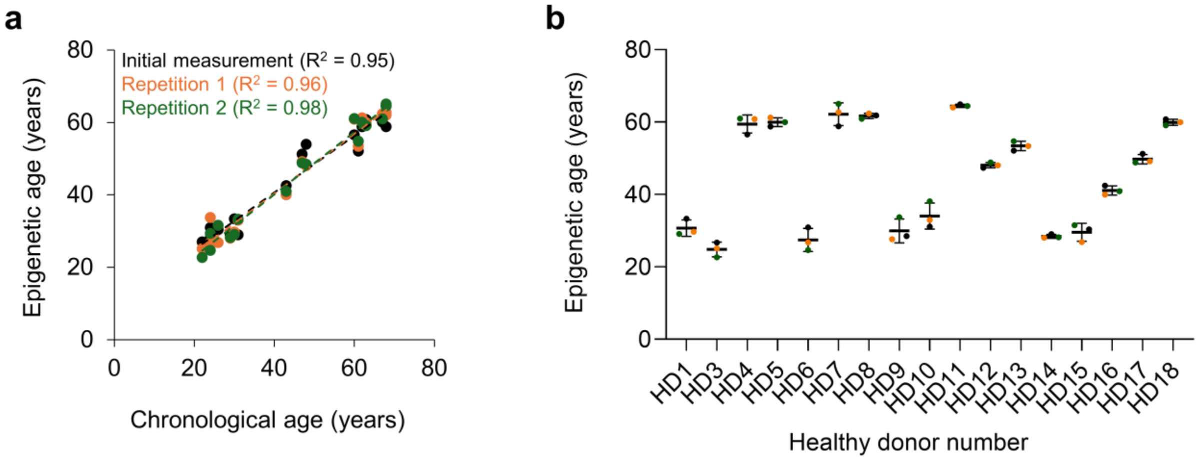

Figure S2: Reanalysis of epigenetic age-predictions in control samples.

To estimate how reproducible epigenetic age-predictions are in repeated measurements, we have reanalyzed the control samples after more than one year (repetition 1 and 2). However, the genomic DNA of HD2 had previously been used up and was no more available. **a**) Comparison of chronological age with epigenetic age showed a high correlation for all three replicates (mean age deviation for repetition 1 = 0.60 years and for repetition 2 = 0.65 years). **b**) Alternative presentation of age-predictions in individual healthy donor samples (HD).

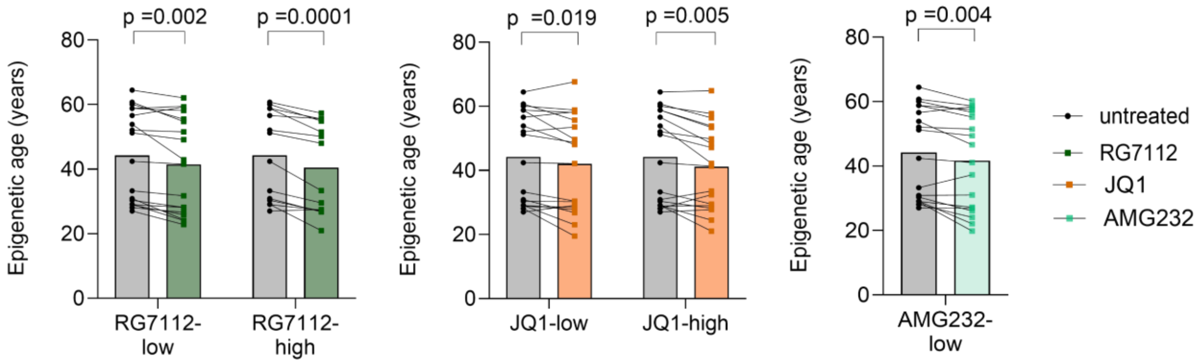

Figure S3: Pair-wise analysis of epigenetic age-predictions upon treatment with senolytic compounds.

While figure 1a depicts the deviation of corresponding epigenetic age predictions in controls and treated samples, we have alternatively performed paired t-test analysis of the epigenetic age predictions. Very similar results were observed and all significant results are depicted. The paired t-tests for all other drugs and concentrations did not reach statistical significance.

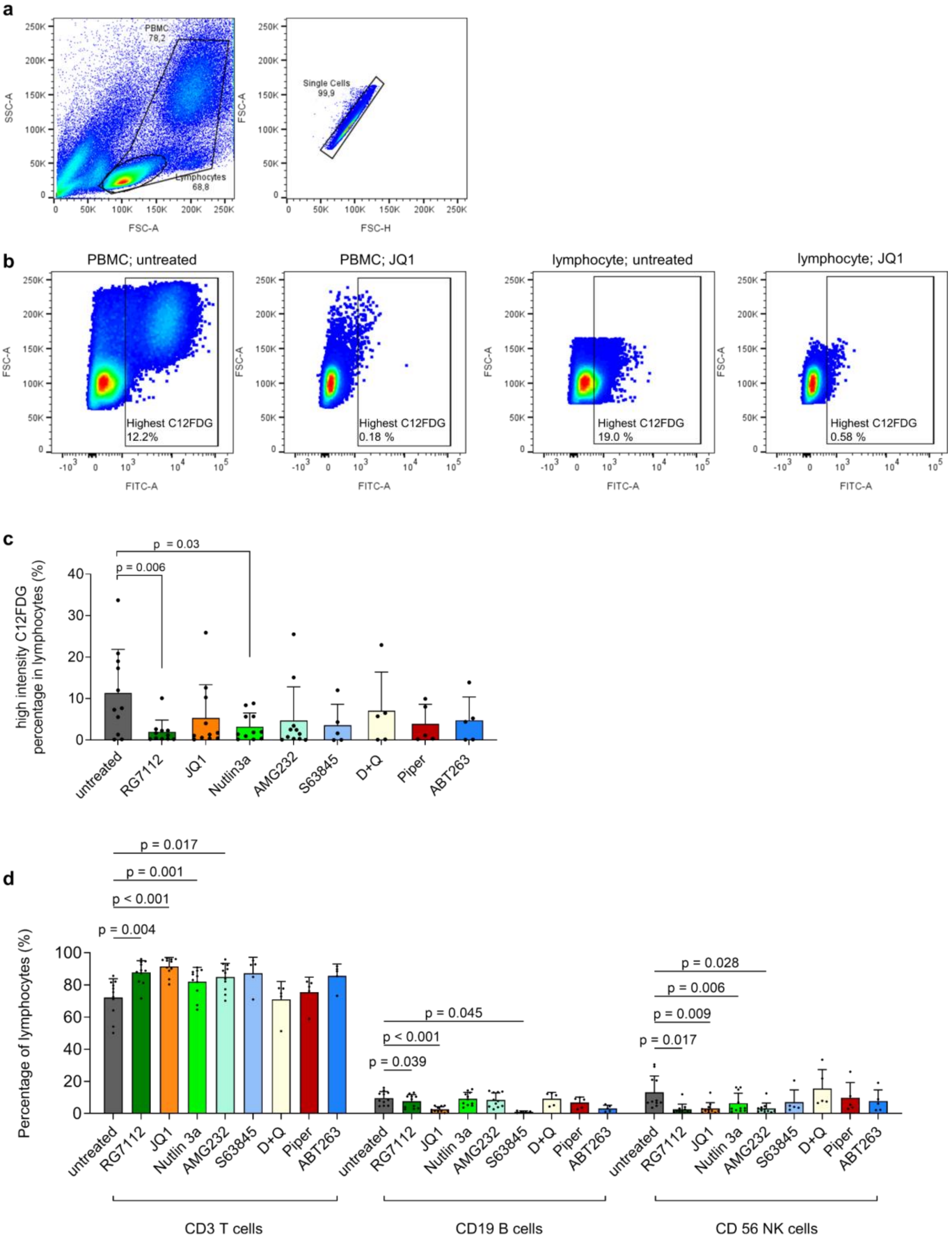

Figure S4: Flowcytometric analysis after treatment with senolytic compounds.

a) Gating strategy for the selection of C12FDG cells is as follows: FSC = forward scatter, SSC = side scatter; gates for PBMCs and lymphocytes are indicated; FSC-A vs FSC-H for doublets exclusion. **b**) High intensity C12FDG-FITC positive cells from a 61-year-old donor. **c**) Percentage of cells with high C12FDG staining in lymphocytes (n = 5 to 11). Two-way ANOVA with multiple comparison was performed to assess statistical significance. **d)** Flow cytometric analysis of the fraction of CD3+ T cells, CD19+ B cells, and CD56+ NK cells without and with senolytic treatment in the lymphocyte compartment. Two-way ANOVA with multiple comparison was performed to assess statistical significance.
